# Remdesivir does not affect mitochondrial DNA copy number or deletion mutation frequency in aged male rats: A short report

**DOI:** 10.1371/journal.pone.0271850

**Published:** 2022-10-26

**Authors:** Allen Herbst, Solbie Choi, Austin N. Hoang, Chiye Kim, Diana Martinez Moreno, Debbie McKenzie, Judd M. Aiken, Jonathan Wanagat

**Affiliations:** 1 Department of Agricultural, Food and Nutritional Sciences, University of Alberta, Edmonton, Canada; 2 Division of Geriatrics, Department of Medicine, UCLA, Los Angeles, California, United States of America; 3 Department of Biological Sciences, University of Alberta, Edmonton, Canada; 4 Veterans Administration Greater Los Angeles Healthcare System, Los Angeles, California, United States of America; University of Parma, ITALY

## Abstract

Remdesivir is a leading therapy in patients with moderate to severe coronavirus 2 (SARS-CoV-2) infection; the majority of whom are older individuals. Remdesivir is a nucleoside analog that incorporates into nascent viral RNA, inhibiting RNA-directed RNA polymerases, including that of SARS-CoV-2. Less is known about remdesivir’s effects on mitochondria, particularly in older adults where mitochondria are known to be dysfunctional. Furthermore, its effect on age-induced mitochondrial mutations and copy number has not been previously studied. We hypothesized that remdesivir adversely affects mtDNA copy number and deletion mutation frequency in aged rodents. To test this hypothesis, 30-month-old male F333BNF1 rats were treated with remdesivir for three months. To determine if remdesivir adversely affects mtDNA, we measured copy number and mtDNA deletion frequency in rat hearts, kidneys, and skeletal muscles using digital PCR. We found no effects from three months of remdesivir treatment on mtDNA copy number or deletion mutation frequency in 33-month-old rats. These data support the notion that remdesivir does not compromise mtDNA quality or quantity at old age in mammals. Future work should focus on examining additional tissues such as brain and liver, and extend testing to human clinical samples.

## Introduction

Coronavirus disease 2019 (COVID-19), caused by the severe acute respiratory syndrome coronavirus 2 (SARS-CoV-2) has been a pandemic for over two years [1, 2]. Older persons are at increased risk for hospitalization or death from COVID-19 [3, 4]. According to a modeling study, the rate of COVID-19 patients hospitalized increased with age: 1% for those 20 to 29 years old, 4% for those 50 to 59 years old, and 18% for those older than 80 years of age [5]. Moreover, the risk for death among individuals 80 years and older is 20-fold higher than among individuals 50 to 59 years old [6]. According to the Center for Disease Control, more than 75% of COVID-19 deaths have been in older patients, including more than a quarter in people aged 85 and older (https://covid.cdc.gov/covid-data-tracker/#datatracker-home).

Remdesivir (Veklury®) is indicated for the inpatient and outpatient treatment of COVID-19 and is standard of care for hospitalized patients with moderate to severe COVID-19. Approximately four million individuals have been hospitalized with COVID as of January 2022. Of these, up to ~50% have received remdesivir [7]. Therefore, approximately two million US patients have received remdesivir thus far–a number that will increase given remdesivir’s recently expanded use as an outpatient therapy. Despite its approval by the Food and Drug Administration (FDA), however, limited clinical data exists pertaining to its safety [8]. Remdesivir has not been evaluated for geriatric use [9]. There is a knowledge gap regarding the safety of drugs such as remdesivir in geriatric populations as individuals >64 years of age are routinely underrepresented in clinical trials [10]. This gap requires research as older individuals are often the primary target population for drugs such as remdesivir.

Remdesivir is a prodrug that is converted *in vivo* into an adenosine nucleoside analogue and subsequently phosphorylated to its triphosphate activated form. The targeted activity of remdesivir is the inhibition of viral RNA-dependent RNA polymerase necessary for the replication of the viral genome [8, 11]. As a nucleoside analogue, remdesivir has the potential for numerous off-target mitochondrial effects that may alter mitochondrial DNA [12, 13]. These off-target effects could impact mitochondrial DNA homeostasis through disruption of mitochondrial polymerases (e.g., DNA polymerase gamma, PrimPol, and mitochondrial RNA polymerase), nucleotide metabolism, mitochondrial respiration, and nucleotide/nucleoside pools. 2’-OH and 3’-OH ribonucleoside analogs such as remdesivir that resemble the building blocks of RNA are expected to have minimal interaction with DNA polymerases. However, mitochondrial DNA polymerase gamma possesses the ability to incorporate ribonucleotides into DNA [14, 15], but with a 1,100-fold to 7,000-fold preference for deoxyribonucleotides [13]. Remdesivir impedes mitochondrial DNA polymerase gamma activity, but stimulates exonucleolytic activity, *in vitro* [16]. PrimPol, another DNA primase/polymerase found in mitochondria is capable of utilizing and extending from ribonucleotides [17, 18]. While remdesivir has minimal effects on the mitochondrial RNA polymerase activity *in vitro* [11, 19], remdesivir had a rate of incorporation of 5.8% relative to ATP when assessing mtRNA polymerase activity [19].

As an ATP-analogue, the active metabolite of remdesivir may participate in numerous unexplored cellular and mitochondrial processes. For example, the active metabolite could affect ATP buffering by creatine kinase and myokinase interfering in muscle metabolism similarly to beta-guanidinoproprionic acid, an analog of phosphocreatine [20, 21]. Remdesivir could also inhibit the adenine nucleotide translocator (ANT), which exchanges ATP and ADP across the mitochondrial inner membrane. Genetic defects in ANT lead to mtDNA deletion mutations and mitochondrial myopathy [22]. The possible impact of remdesivir on any of these mitochondrial processes has implications for mtDNA replication, copy number, and mutation frequency.

The reported mitochondrial effects of remdesivir vary widely. Direct *in vitro* effects of remdesivir on isolated pig brain mitochondria showed little or no effect on mitochondrial respiration [23]. However, remdesivir induced persistent mitochondrial changes including mitochondrial fragmentation, reduced redox potential, and suppressed mitochondrial respiration in an *in vitro* human cardiac stem cell model [24]. Remdesivir decreased mitochondrial respiratory gene expression, ATP production, and mitochondrial oxidation in human intestinal and liver cell lines [25 Preprint]. With respect to remdesivir’s effect on the mitochondrial genome, remdesivir did not alter mitochondrial DNA copy number in human liver cells or skeletal muscle fibroblast cells [26], but increased mtDNA copy number in human neonatal dermal fibroblasts [16] and HepG2 cells [19]. There have been fewer *in vivo* studies, but remdesivir treatment in rhesus monkeys resulted in a significant loss of mtDNA in an unspecified tissue [27]. These studies suggest cell and tissue specific effect of remdesivir that may impact the quality or quantity of mitochondrial DNA. The *in vivo* effects of remdesivir on mitochondrial DNA copy number and mutations in aged animals is unknown. This is especially relevant as remdesivir is administered predominantly to older COVID-19 patients.

The expanded use of remdesivir has led to the identification of important side effects including cardiac and renal effects. In the cardiovascular system, remdesivir increased the risk of bradycardia (Reporting Odds Ratio 1.63) and hypotension [28]. In the renal system, remdesivir treatment can cause acute renal failure, with 65-74-year-old old adults being at the highest risk [29, 30]. The role for mitochondria in these side effects is unclear but is likely related age-induced changes in these tissues and the reliance of heart and kidney on oxidative metabolism. Musculoskeletal side effects have not been reported for remdesivir treatment of COVID-19 patients.

**Fig 1:**
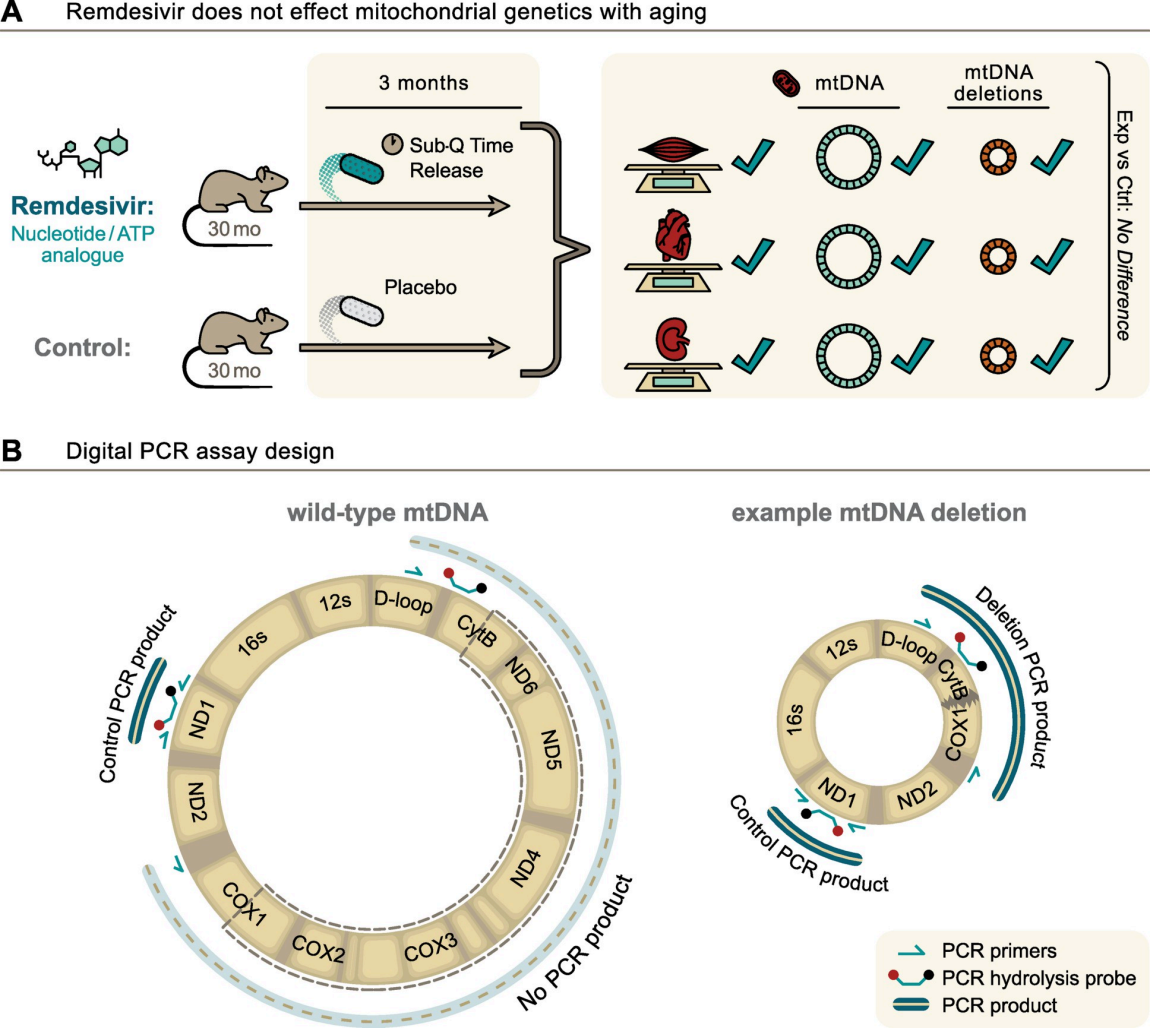
Graphical abstract of the study (A) and design of digital PCR assay (B). Primers and probes are not drawn to scale. Primers and probe used to quantitate mtDNA deletion mutations flank the mitochondrial major arc to detect age-induced mtDNA deletion mutations. In the wild-type genome in the left panel, the major arc-flanking primers are too far apart for amplification. In the right panel, an example mtDNA deletion mutation has brought the major arc-flanking primers within range for amplification of a PCR product.

MtDNA copy number and deletion frequency predict age and some measures of physical function in older adults [31, 32] and may be metrics of biological age. We hypothesized that remdesivir would negatively affect mtDNA quality by altering copy number and increasing mitochondrial mutation frequency in aged animals. To test this hypothesis, we measured mtDNA copy number and deletion mutation frequency in heart, kidney, and skeletal muscle from 33-month-old male rats treated with remdesivir for 3 months. Remdesivir had no effect on either mtDNA copy number or mutation frequency at old age in this model, indicating that remdesivir does not negatively impact *in vivo* mitochondrial DNA quality and quantity in aged animals (**Fig 1**).

## Materials and methods

### Animals, remdesivir treatment, and tissue preparation

This study was carried out in accordance with the recommendations in the NIH Guide for Care and Use of Laboratory Animals and the guidelines of the Canadian Council on Animal Care using protocols approved by the Institutional Animal Care and Use Committees at UCLA and the University of Alberta. Thirty-month-old male Fischer 344 x Brown Norway F1 hybrid rats were obtained from the NIA Aging Rodent Colony. Remdesivir (MW 602.6) was purchased from VulcanChem (Pasadena, CA) and its authenticity was confirmed by mass spectrometry (S1 File). Drug delivery was via the subcutaneous implantation of a ninety-day time-release pellet containing 200 mg of remdesivir (Innovative Research of America, Sarasota, FL). The dose delivered over the 90 days was approximately 4.25 mg/kg/day. To determine a dosage for rats, we started with the human dose for remdesivir which is 1.25 mg/kg. Using the body surface area approach [33] the equivalent rat dose would be 7.8 mg/kg. We were limited to 4.25 mg/kg/day because of restrictions on the pellet size and available amounts of remdesivir during the pandemic. Rats were housed on a 12-hour light/dark cycle and fed standard chow. Control rats were implanted with a placebo pellet prepared from the time-release matrix only. Animals were euthanized by carbon dioxide asphyxiation followed by exsanguination. Tissues were dissected from the rats, weighed, and flash frozen in liquid nitrogen, and stored at -80°C.

### DNA isolation

Rat heart, kidney, and quadriceps muscle were ground to a powder using a mortar and pestle under liquid nitrogen. Total DNA was extracted using proteinase K digestion with SDS and EDTA, phenol/chloroform extraction, and ethanol precipitation. Total DNA was resuspended in 10 mM Tris-EDTA buffer, pH 8. Total DNA quality and quantity was assessed using spectrophotometry at A230, A260, and A280 (ThermoScientific Nanodrop 2000 Spectrophotometer), fluorometry (ThermoFisher Qubit 2.0 Fluorometer) and agarose gel electrophoresis.

### MtDNA copy number and mtDNA deletion frequency by digital PCR

A 5-prime nuclease cleavage assay and droplet-based digital PCR (dPCR) were used to quantitate copy numbers for nuclear DNA (nDNA), total mtDNA, and mtDNA deletion mutations. We used a digital PCR approach for these measures. Digital PCR is not dependent on or affected by the amplification efficiency of the PCR primers or other confounders of quantitative PCR methods [34]. Specific primer/probe sets were used for each target as previously described [35] and illustrated in **Fig 1B**. MtDNA deletion frequency is the proportion of mutant molecules per wild-type mtDNA molecules. MtDNA copy number and mtDNA deletions per 100 nuclei are values normalized to the single copy nuclear gene in the rat, Unc13 [36]. DPCR quantitation of all samples and all targets was performed on coded samples. Blinding was removed following data collection.

### Statistical analysis

All data are presented as means ± SEM. Data were tested for a normal distribution. Student’s t-test was used to compare differences between treatment and control groups. Chi-squared test was used to determine differences in survival frequency. One-way analysis of variance was used to test statistical differences between tissues. Prism (Version 7.05, GraphPad Software) was used for all statistical analyses.

## Results

### Husbandry and morphometric measures following remdesivir treatment

**Fig 2:**
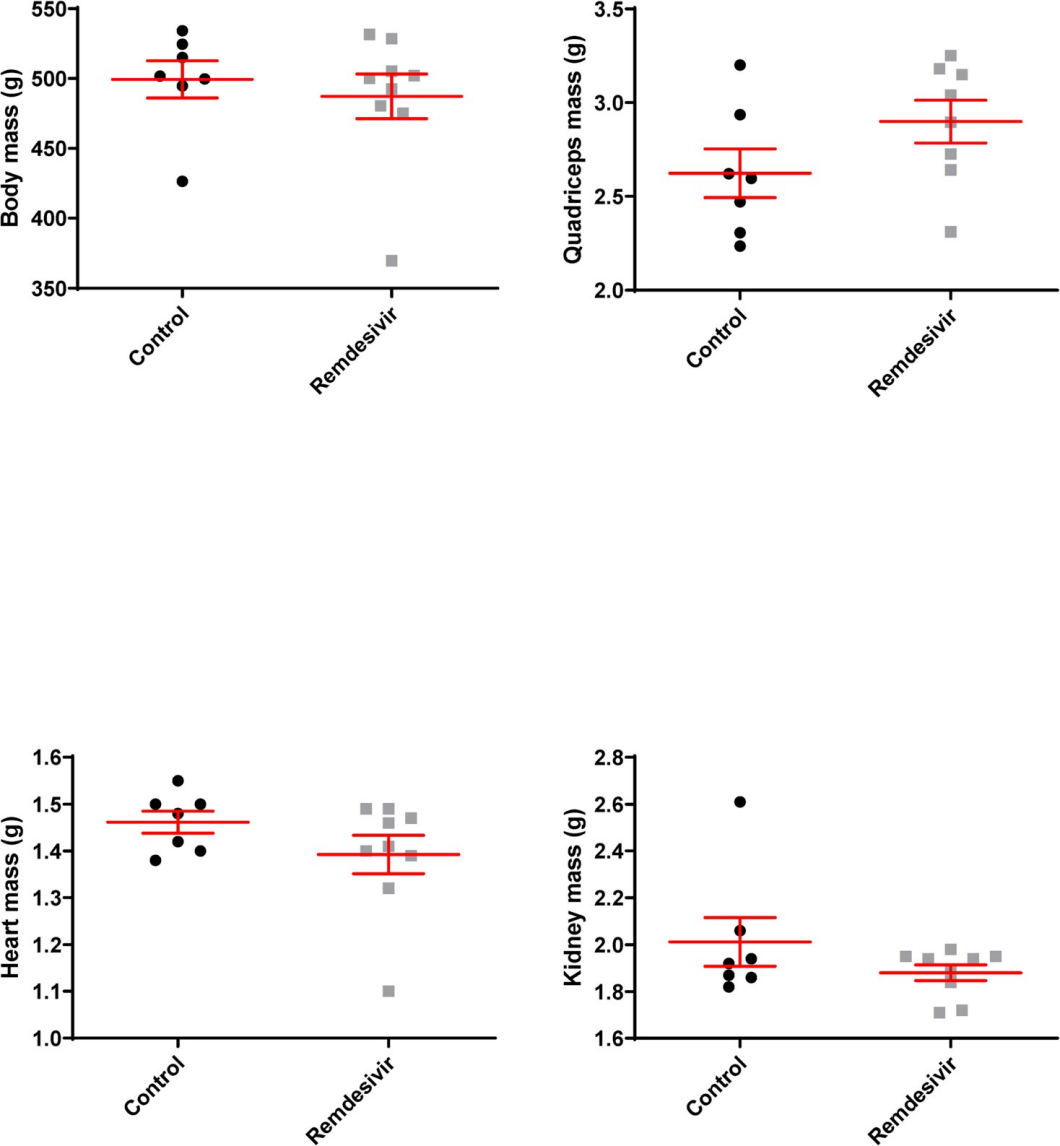
Effects of three months of remdesivir treatment on body, heart, kidney and quadriceps mass in hybrid rats. Rats were weighed at 33 months of age before they were sacrificed. Whisker plots denote mean and SEM. Black circles denote control rats, grey squares denote remdesivir-treated rats. N = 7–9 per experimental group.

Ninety days of subcutaneous remdesivir treatment starting at 30 months in male F344BN F1 hybrid rats had no adverse effects on food consumption as noted by observation and lack of difference in body weights before and after treatment between control and treated rats (S1 Fig). The mean lifespan of male F344BNF1 rats is 34 months [37] and rat survival was not affected by remdesivir treatment with three control and two remdesivir-treated rats reaching a point where rat body condition scoring indicated that death was imminent and animals were culled during the 90 days of the experiment (Chi squared = 0.582). Body, heart, kidney, and muscle weights also were not affected by the remdesivir treatment (**Fig 2**).

### Measures of mtDNA copy number and deletion mutation frequency following remdesivir treatment

**Fig 3:**
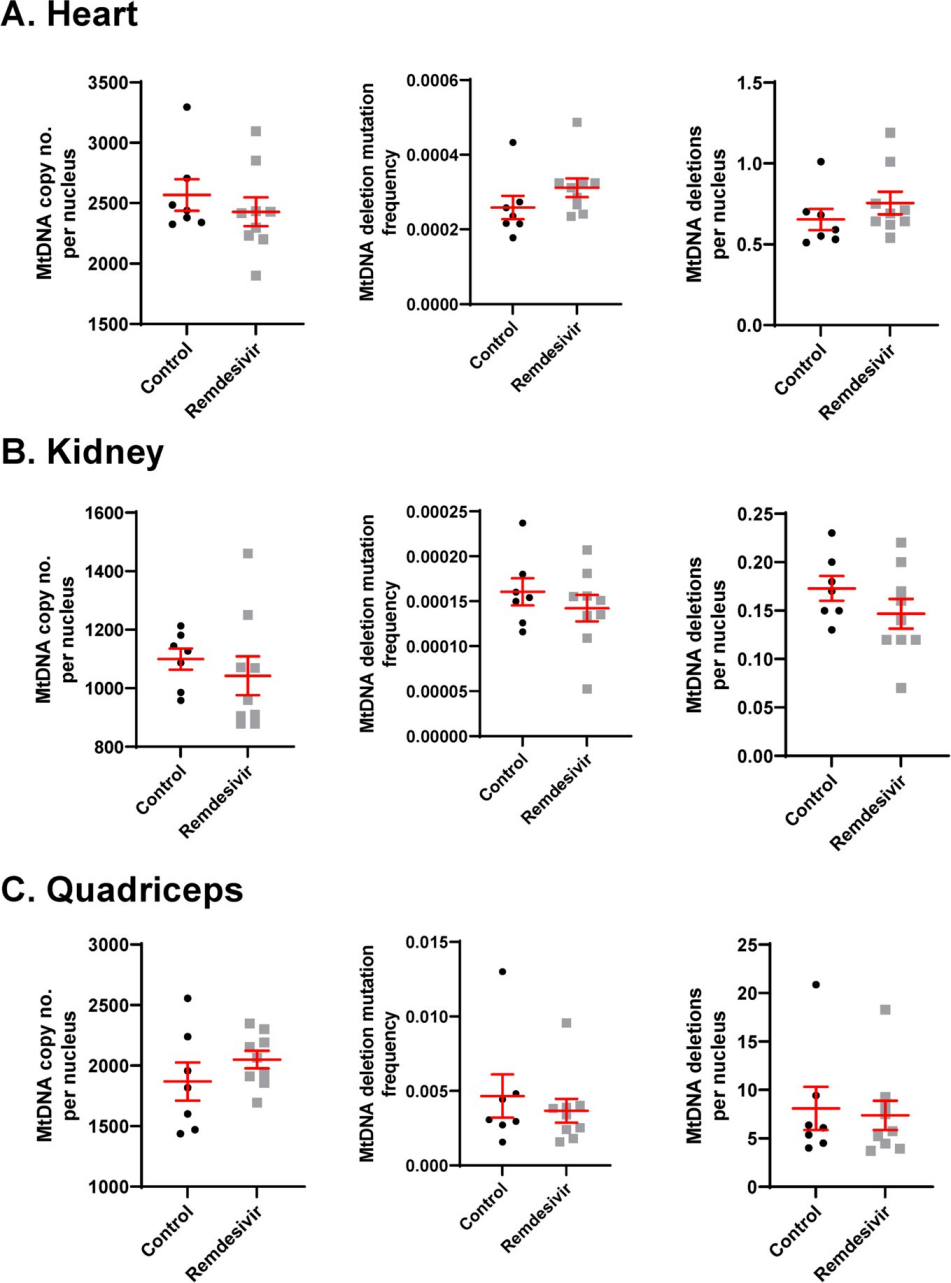
Effects of three months of remdesivir treatment on mtDNA copy number, deletion frequency, and deletions per nucleus in 33-month-old hybrid rats. A. heart, B. kidney, and C. quadriceps muscle. Deletion frequency and deletions per nucleus correspond to interrogation of the major arc of the mitochondrial genome as demonstrated in Fig 1B. Whisker plots denote mean and SEM. Black circles denote control rats, grey squares denote remdesivir-treated rats. N = 7–9 per experimental group.

Similar to the husbandry and morphometric outcomes, mtDNA copy number and deletion mutation frequency were not affected by 90 days of remdesivir treatment (**Fig 3**). For the 33-month-old control versus treated rats, the average mtDNA copy number per diploid nucleus was 2567 versus 2428 (p = 0.45, 95% CI -519.6 to 242.1), 1100 versus 1043 (p = 0.46, 95% CI -221.6 to 107.7), and 1869 versus 2050 (p = 0.32, 95% CI -213.3 to 575.5) for heart, kidney, and quadriceps, respectively. To assess the cellular burden of mtDNA deletion mutations, we calculated the number of deletion mutations per diploid nucleus and found no effects of remdesivir. MtDNA deletion mutation frequency in control versus treated rats was 2.6x10^-4^ versus 3.1x10^-4^ (p = 0.21, 95% CI -3.433e-5 to 1.399e-4), 1.6x10^-4^ versus 1.4x10^-4^ (p = 0.40, 95% CI -6.337e-5 to 2.704e-5) and 4.7x10^-3^ versus 3.7x10^-3^ (p = 0.57, 95% CI -4.697e-3 to 2.731e-3) for heart, kidney, and quadriceps, respectively. MtDNA copy number and deletion mutation frequency differ greatly between heart, kidney, and skeletal muscle at 33 months. Heart mtDNA copy number was 1.70-fold higher than kidney and 1.37-fold higher than quadriceps, while the deletion mutation frequency in skeletal muscle was 18-fold higher than heart and 29-fold higher than kidney.

## Discussion

To our knowledge, this is the first study examining the impact of remdesivir on mtDNA metrics in aging rodents. As mitochondrial dysfunction is a conserved phenotype of aging with accessible and sensitive biomarkers, we investigated the effect of remdesivir on mitochondrial genetics, i.e., copy number and mtDNA deletion mutation frequency, in aged rats. We found that 90 days of remdesivir treatment did not alter mitochondrial copy number or deletion mutation frequency in 33-month-old male F333BNF1 rats. Furthermore, in the aged rats, we did not observe any effect on body weight, food consumption, or organ weights. Our data adds to the limited information available on remdesivir’s effect on mitochondria, especially in older individuals. Also, unlike other studies that focus on a single tissue, our study examined remdesivir’s impact on mitochondrial quality in multiple organ/tissue systems, specifically, heart, kidney, and skeletal muscle. Our findings indicate that the use of remdesivir to treat COVID in aging patients is not detrimental to their mitochondrial indices.

Remdesivir’s *in vivo* effects on the mtDNA polymerase and its subsequent impact on mitochondrial genetics are just beginning to be understood. Our data do not support the hypothesis that remdesivir affects mtDNA *in vivo*, despite the varied *in vitro findings* and *in vivo* findings in young animals [16, 26]. Moreover, although IV remdesivir in rhesus macaques, at a therapeutic concentration of 1μM remdesivir, showed no significant reduction in mtDNA copy number, a higher therapeutic level (2 μM) resulted in a 26% decrease in mtDNA copy number [27]. In contrast, when Bjork and Wallace [26] assessed the dose-dependent effect of remdesivir on mitochondrial DNA replication by exposing human hepatoma HepG2/C3A cells to increasing concentrations of remdesivir (0.1 to 10 μM) for 24 h and 48 h, they did not observe changes in mtDNA copy number. Given the pharmacokinetics of remdesivir in rats [38], our daily dose of 4.25 mg/kg of remdesivir is comparable to the human dose of 1.25 mg/kg.

The effects of remdesivir on the accumulation of mtDNA deletion mutations has received very little attention. In human neonatal dermal fibroblasts, remdesivir treatment did not induce the 4977 or “common” mtDNA deletion mutation [16]. Our data from remdesivir treatment in aged rats indicate that remdesivir also does not contribute to age-induced mitochondrial genetic structural rearrangements and reassuring of remdesivir safety in older individuals.

Little is known about the effect of remdesivir on mitochondrial transcription. Bjork and Wallace [26] studied the dose-dependent effect of remdesivir on mitochondrial gene expression *in vitro* and found no evidence that remdesivir interferes with gene transcription. In a separate study, when HepG2 (liver) and HT-29 (intestinal) cells, treated with remdesivir for 8 and 24 h (in contrast to 24 and 48 h for Bjork et al), were analyzed by RNAseq, there was a decrease in the expression of genes involved in mitochondrial respiration and an observation that remdesivir decreases cellular ATP [25 Preprint]. They hypothesized that this reduction in ATP was, at least in part, due to a reduction in mitochondrial gene expression. The discrepancy in these two studies should be tested, using aged rodents, in transcriptomic studies such as RNAseq. Aged rodent studies would have the added advantage of utilizing *in vivo* samples, compared to the aforementioned *in vitro* studies, providing a clearer and more accurate picture. Additionally, an aged rodent model would be conducive to testing remdesivir for side effects that might be expected in older adults.

Unlike human COVID-19 patients who are administered remdesivir via intravenous infusion, the aged rats in this study were given remdesivir in the form of a subcutaneously implanted time-release tablet. The remdesivir treatment in our study was considerably longer– 90 days–as opposed to a 5-10-day course in humans. We would, therefore, predict more pronounced side effects in the aged rats treated with remdesivir, compared to those seen in humans. This is not what we observe. Our results demonstrate that remdesivir is not detrimental to mitochondrial quality and quantity in an appropriately aged animal model. We examined rat heart, kidney, and skeletal muscle, but remdesivir has also been reported to have hepatic side effects [39]; we did not examine liver tissue for mtDNA effects. For mtDNA copy number and deletion frequency, our minimal effects of interest would be increases or decreases greater than 1.5-fold. These effects of interest are based on previous studies showing that skeletal muscle mtDNA copy number is increased by more than 2-fold by interventions such as exercise [40] and deletion mutation frequency is increased by ~2-fold with other drug treatments that have significant physiological effects in aged rats [41]. As our effects of interest fall outside the 95% confidence intervals for our measurements of mtDNA copy number and deletion frequency with remdesivir treatment, we interpret these negative results to be meaningful [42].

In summary, our data demonstrate that remdesivir does not compromise mitochondrial copy number or mtDNA deletion mutation frequency in heart, kidney, and skeletal muscle in aged rodents. In addition to testing its impact on other relevant tissues like brain and liver, future work should focus on examining the effect of remdesivir on *in vivo* mitochondrial transcription. Finally, because rodent studies are unable to completely predict human outcomes, similar studies should be extended to clinical samples of human COVID-19 patients who received remdesivir treatment.

## Supporting information

S1 Fig Initial and final body mass. Rats were weighed at 30 months of age before starting placebo or remdesivir treatment and at 33 months of age before they were sacrificed. Whisker plots denote mean and SEM. Black circles denote control rats, grey squares denote remdesivir-treated rats. N = 7–9 per experimental group.
(TIF)

S1 File Mass spectrometry confirmation of remdesivir. (PDF)

S2 File Supporting data. All data necessary to replicate study findings.
(XLSX)
