# Supplementary figures and images for: Remdesivir does not affect mitochondrial DNA copy number or deletion mutation frequency in aged male rats: A short report
Source: PLoS One. 2022 Oct 26;17(10):e0271850. doi: 10.1371/journal.pone.0271850 (PMC9605030; doi:10.1371/journal.pone.0271850)

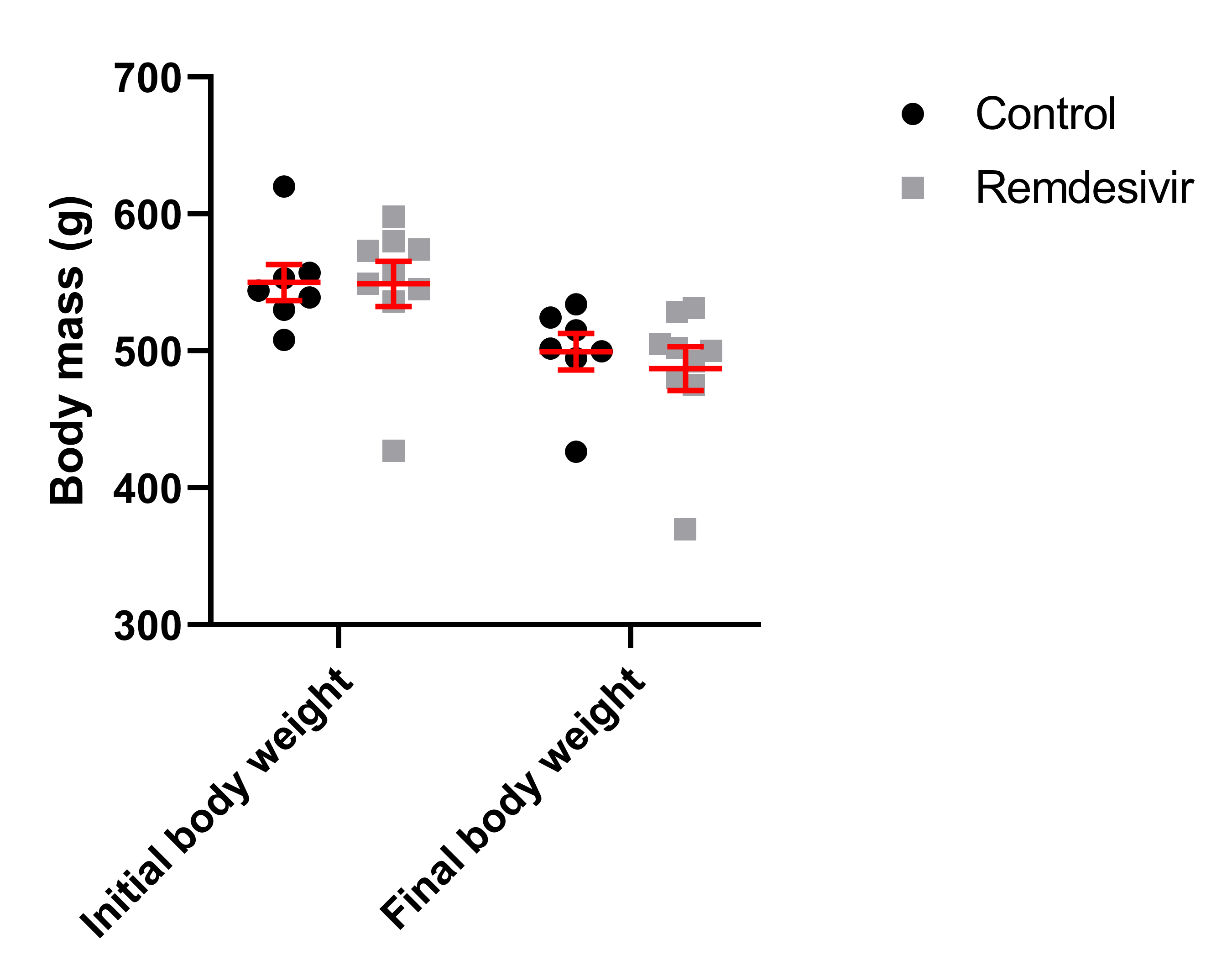

Supplement: S1 Fig — Rats were weighed at 30 months of age before starting placebo or remdesivir treatment and at 33 months of age before they were sacrificed. Whisker plots denote mean and SEM. Black circles denote control rats, grey squares denote remdesivir-treated rats. N = 7–9 per experimental group. (TIF) [file pone.0271850.s001.tif]
