# Supplementary material for: Remdesivir does not affect mitochondrial DNA copy number or deletion mutation frequency in aged male rats: A short report
Source: PLoS One. 2022 Oct 26;17(10):e0271850. doi: 10.1371/journal.pone.0271850 (PMC9605030; doi:10.1371/journal.pone.0271850)
Supplement: S1 File — (PDF) [file pone.0271850.s002.pdf]

Department of Chemistry Mass Spectrometry Laboratory

**Name**	D.M. Moreno, J. Aiken<br>Walkup method: 'C8_acid'	**Sample Name**	Remdesivir
**Data Filename**	Remdesivir_1385.D	**Instrument Name**	6130 LCMS
**Position**	Vial 10	**Operator**	Administrator
**Acq Method**	C8_Acid_COL2.M	**DA Method**	1_LCMS_SQ.m

User Chromatograms

Fragmentor Voltage 0 **Collision Energy** 0 **Ionization Mode** ESI

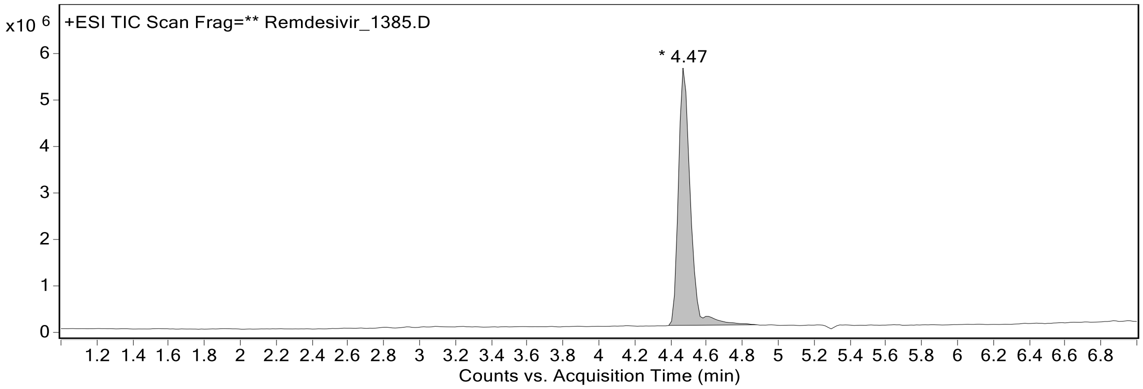

Integration Peak List

Peak	Start	RT	End	Height	Area	Area %	AreaSum%
1	4.39	4.47	4.88	5523233	25706631	100	100

Fragmentor Voltage 0 **Collision Energy** 0 **Ionization Mode** ESI

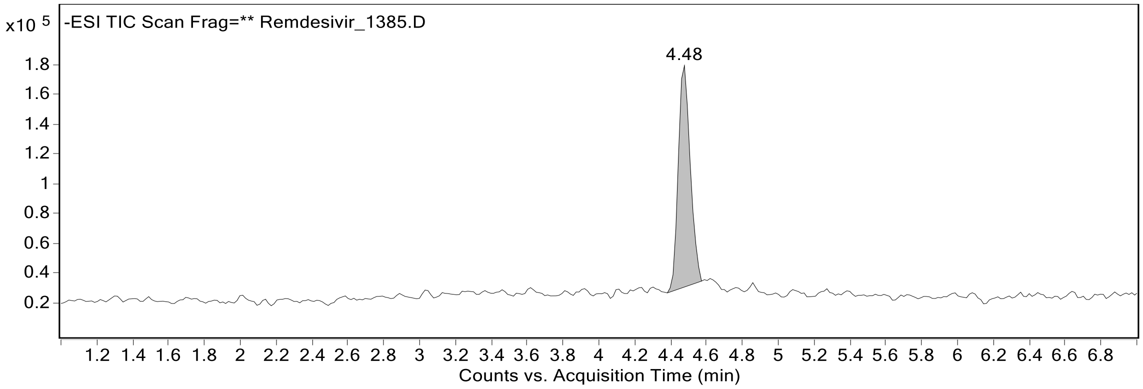

Integration Peak List

Peak	Start	RT	End	Height	Area	Area %	AreaSum%
1	4.38	4.48	4.57	148516	695753	100	100

Fragmentor Voltage 0 **Collision Energy** 0 **Ionization Mode** ESI

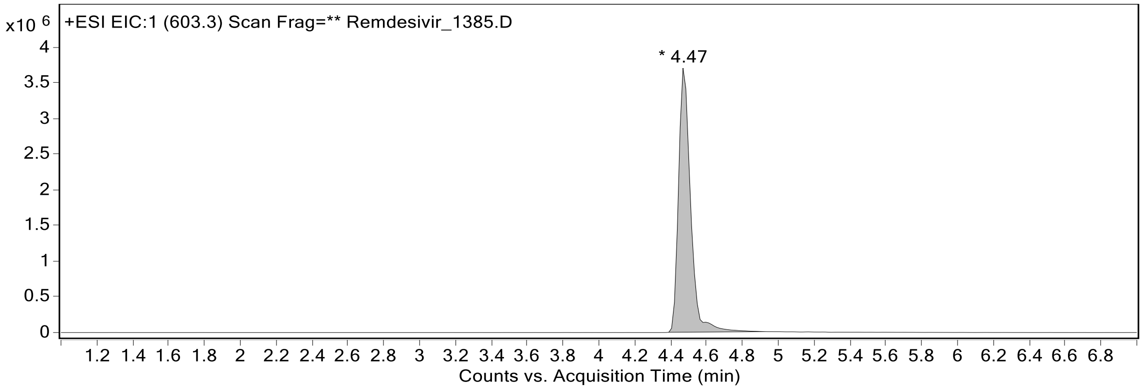

Integration Peak List

Peak	Start	RT	End	Height	Area	Area %	AreaSum%
1	4.39	4.47	4.93	3705028	17386891	100	100

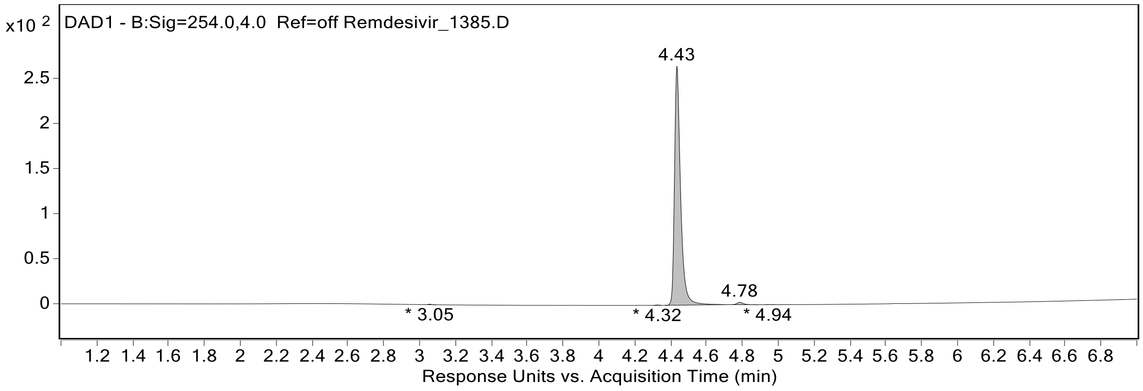

Integration Peak List

Peak	Start	RT	End	Height	Area	Area %	AreaSum%
1	3.03	3.05	3.09	0.48	0.56	0.09	0.09
2	4.3	4.32	4.35	0.46	0.56	0.09	0.09
3	4.37	4.43	4.72	264.97	627.06	100	98.72
4	4.72	4.78	4.89	2.64	6.69	1.07	1.05
5	4.92	4.94	4.95	0.15	0.17	0.03	0.03
6	4.95	4.97	4.99	0.15	0.16	0.03	0.03

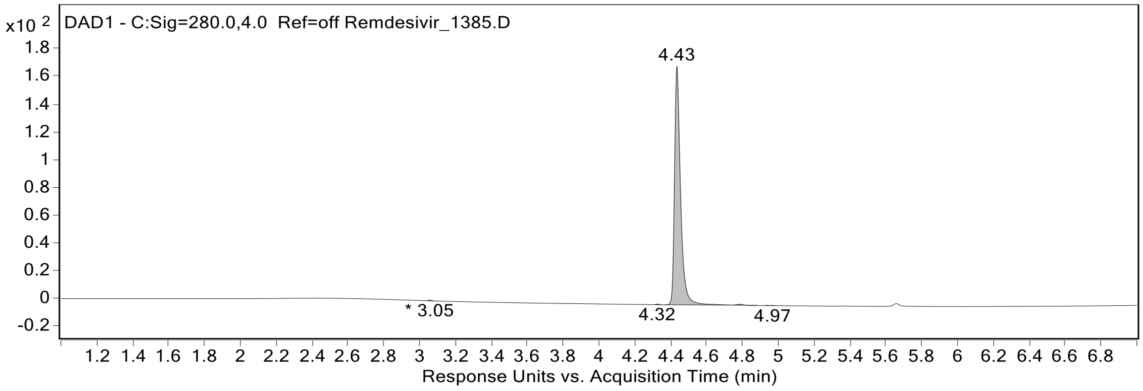

Integration Peak List

Peak	Start	RT	End	Height	Area	Area %	AreaSum%
1	3.03	3.05	3.08	0.46	0.54	0.13	0.13
2	4.3	4.32	4.35	0.45	0.54	0.13	0.13

3	4.37	4.43	4.73	171.87	404.36	100	99.26
4	4.74	4.78	4.88	0.66	1.62	0.4	0.4
5	4.92	4.94	4.95	0.14	0.16	0.04	0.04
6	4.95	4.97	4.99	0.15	0.16	0.04	0.04

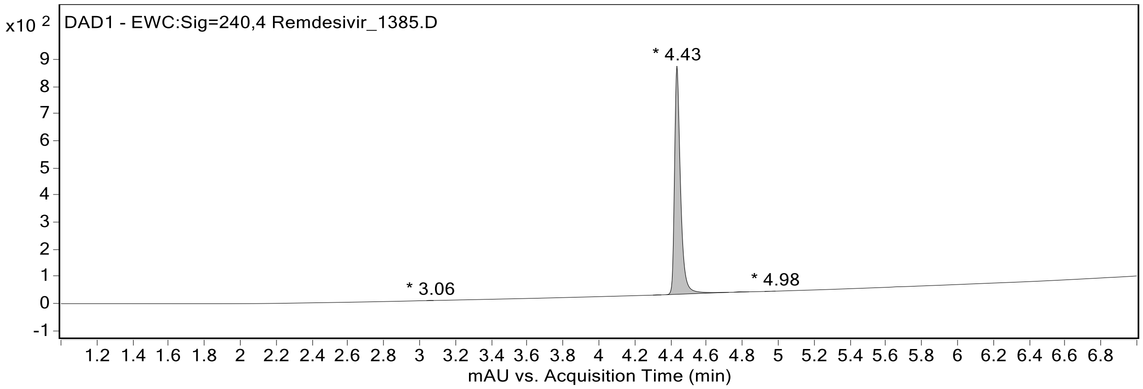

Integration Peak List

Peak	Start	RT	End	Height	Area	Area %	AreaSum%
1	3.04	3.06	3.08	0.43	0.607	0.03	0.03
2	4.3	4.33	4.35	0.39	0.659	0.03	0.03
3	4.37	4.43	4.72	843.12	1975.357	100	99.79
4	4.75	4.79	4.84	1.04	2.458	0.12	0.12
5	4.92	4.95	4.95	0	0.248	0.01	0.01
6	4.95	4.98	4.98	0	0.228	0.01	0.01

User Spectra

Fragmentor Voltage	Source Type
0	ESI

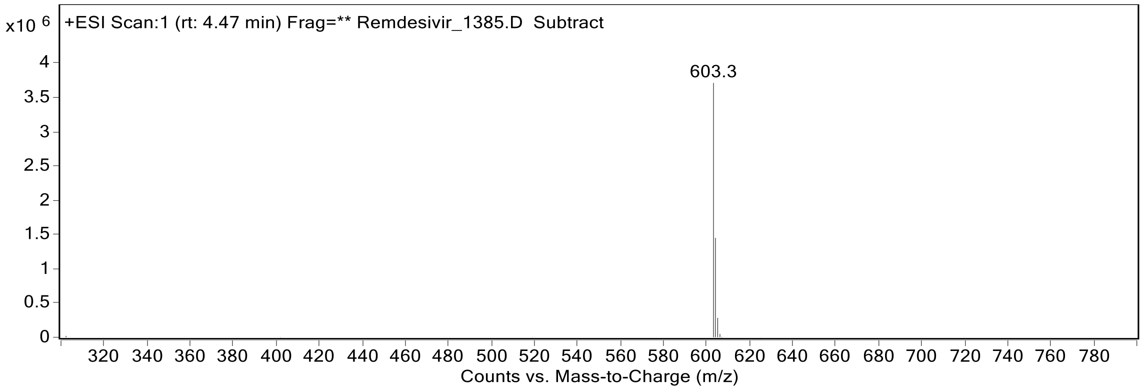

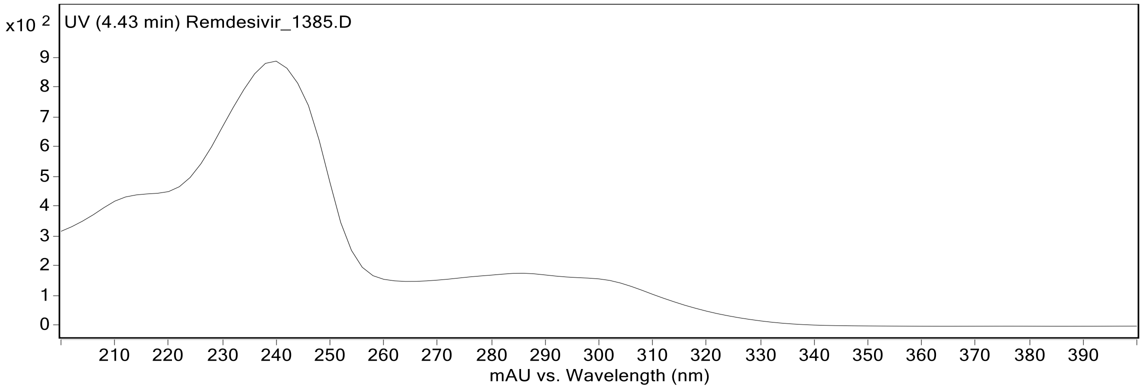

--- End Of Report ---
